# Different brain structures associated with artistic and scientific creativity: a voxel-based morphometry study

**DOI:** 10.1038/srep42911

**Published:** 2017-02-21

**Authors:** Baoguo Shi, Xiaoqing Cao, Qunlin Chen, Kaixiang Zhuang, Jiang Qiu

**Affiliations:** 1 Beijing Key Laboratory of Learning and Cognition and Department of Psychology, Capital Normal University, Beijing 100048, China; 2 Key Laboratory of Cognition and Personality (SWU), Ministry of Education, Chongqing 400715, China; 3 School of Psychology, Southwest University, Chongqing 400715, China

## Abstract

Creativity is the ability to produce original and valuable ideas or behaviors. In real life, artistic and scientific creativity promoted the development of human civilization; however, to date, no studies have systematically investigated differences in the brain structures responsible for artistic and scientific creativity in a large sample. Using voxel-based morphometry (VBM), this study identified differences in regional gray matter volume (GMV) across the brain between artistic and scientific creativity (assessed by the Creative Achievement Questionnaire) in 356 young, healthy subjects. The results showed that artistic creativity was significantly negatively associated with the regional GMV of the supplementary motor area (SMA) and anterior cingulate cortex (ACC). In contrast, scientific creativity was significantly positively correlated with the regional GMV of the left middle frontal gyrus (MFG) and left inferior occipital gyrus (IOG). Overall, artistic creativity was associated with the salience network (SN), whereas scientific creativity was associated with the executive attention network and semantic processing. These results may provide an effective marker that can be used to predict and evaluate individuals’ creative performance in the fields of science and art.

Creativity has been viewed as the ability to produce original, unusual, flexible, and valuable ideas or behaviors that override an established mental habit^1^. The study of creativity is undoubtedly important for the future of humans because creativity is the driver of social progress and affects all aspects of human life^2^. Runco^3^ even stated that as the society around us becomes increasingly complex, creativity plays a more crucial role than ever before.

The prior study suggests that the anterior cingulate cortex (ACC) is involved in creativity^4^. The right ACC is one of the core regions of the salience network (SN)^5^, and Seeley *et al*.^6^ suggested that the SN reacts to behaviorally salient events and plays an important role in cognitive processes, such as the initiation of cognitive control^7^, the maintenance and execution of tasks^8^, and the ranking of behavioral responses^9^. While some studies have emphasized the outstanding role of the ACC in creativity, other studies have shown that the left inferior parietal lobule (IPL)^10^, right angular gyrus^10^, the dorsolateral prefrontal cortex (DLPFC)^4^ and left middle temporal gyrus (MTG)^11^ are also involved in creativity. According to Beaty *et al*.^12^, creativity involves a distributed network, including the left precuneus, right posterior cingulate cortex (PCC), and bilateral IPL, which are regions within the default mode network (DMN)^12^; the right DLPFC, which is a core region of the executive control network (ECN); and the right ACC and bilateral insula, which are core regions of the SN^5^. Also, they suggested that the distributed network included a few of significant clusters which belong to the temporal lobes (e.g., the MTG bilaterally), regions are associated with semantic and episodic memory retrieval. Based on the findings of these prior studies, there is no clear consensus about the neural basis of creativity, and one could even infer that creativity may involve several important networks, including the ECN, SN and DMN.

Real-life human activities can be divided into two main fields: science and art. In fact, the boundary between artistic creativity and scientific creativity occurred due to changes in the systems and structure of education. Over the past century, European government agencies emphasized specialization and founded two types of educational funds: artistic and scientific^13^. In China, college students must take a science elective course and a literature elective course in addition to their required courses. This classification of courses implicitly drives people into two ways of thinking: artistic and scientific. Creativity is also divided into artistic creativity and scientific creativity, each with its own characteristics. Is this difference reflected neurally? Here, the present article will review related studies of artistic and scientific creativity.

Early studies of the relationship between artistic creativity and brain structure focused primarily on brain injury. Exploring the relationship between injured brain regions and patients’ behavior can enable indirect inferences about the brain structures involved in creativity^14^. Numerous studies of brain injury have revealed that artistic creativity is closely associated with the right lateral prefrontal cortex^15^, the right neocortex^16^, the left ventral thalamus^17^, bilateral frontal temporal lobe, anterior hippocampus, bilateral temporal pole, inferior temporal gyrus, MTG and left amygdala^18^. These results are inconsistent. With the generation of new technology, more attention has been paid to gray and white matter. Schlegel *et al*.^19^ found that art students became more creative compared with the control group via the reorganization of prefrontal white matter. Bashwiner *et al*.^20^ suggested that musically creative people (as indicated by self-report) had greater cortical surface area or volume in regions associated with domain-specific higher-cognitive motor activity and sound processing, these regions including dorsal premotor cortex, supplementary and pre-supplementary motor areas, and planum temporale. Chamberlain *et al*.^21^ revealed that artistic training was related to increased gray matter density in the right precuneus. Taken together, prior studies have reached conflicting conclusions, and more studies are needed to be conducted to explore the neural basis of artistic creativity.

Throughout the investigation of scientific creativity, studies of only scientific creativity have been rare. The majority of studies of the neural mechanisms of scientific creativity are based on anatomy. Albert Einstein (1879.3.14 ~ 1955.4.18) is regarded as one of the most creative scientific geniuses in human history, and researchers have tried to explore the neural mechanisms of scientific creativity by studying the anatomy of his brain. Diamond *et al*.^22^ found that Einstein’s brain contained more nerve cells and glial cells than those of normal individuals. Anderson and Harvey^23^ indicated that the total weight of Einstein’s brain was less than that of the average adult male’s brain. And they found that the cortex in Brodmann area 9 was thinner in Einstein’s brain than in those of the control group, but the density of nerve cells was higher. To some extent, this result is consistent with previous results such as those of Diamond. Witelson *et al*.^24^ found that in Einstein’s brain, the posterior ascending branch of the lateral fissure in both the left and right hemispheres joined the postcentral sulcus. So, Einstein’s brain had no parietal operculum. They also found that Einstein’s parietal cortex was 15% wider than that of the brain’s in the control group, and the back end of the lateral fissure was widest in Einstein’s brain^24^. Some researchers concluded that these unique features were neuroanatomical correlates of a high degree of creativity. Cognitive neuroscience studies have also shown that the parietal lobe plays an important role in mathematical thinking and vision^24^,^25^. The uniqueness of Einstein’s parietal lobe may be responsible for his high creativity. Anatomical studies of the brains of other mathematicians and physicists also support the conclusion that specific parietal lobe characteristics are associated with creativity^24^,^25^,^26^. In recent years, several neuroimaging and electrophysiology studies have explored the neural basis of scientific creativity. The results of these studies indicated that scientific creativity is closely associated with the frontal lobe, parietal lobe and cingulate gyrus^27^. Hao *et al*.^28^ found that the MTG and the middle occipital gyrus (MOG) were related to scientific problem solving, while Tong *et al*.^29^ found that the MOG, right PCC, and left MFG were related to scientific problem solving. However, overall, fewer studies of the neural basis of scientific creativity have been conducted, and the neural basis of scientific creativity remains elusive.

Haier *et al*.^30^ concluded that regional GMV is the basis of intellectual abilities, and other authors have suggested that structural imaging of regional GMV can provide information about creativity^31^. Based on the fact that the results of previous studies cannot reach an incontestable conclusion, and there is no study to investigate the brain structure between artistic and scientific creativity so far. The purpose of the current study was to reveal differences in regional GMV between artistic and scientific creativity. Through this study, we sought to identify differences in the neural basis of artistic and scientific creativity and to provide a foundation for future research. Specifically, the participants in the present study underwent MRI scans and psychological tests, including a creative achievement questionnaire and intelligence testing. The creative achievement questionnaire was used to assess individual artistic creativity and scientific creativity, while GMV was used as a measure of individual brain structure. We believe the results will provide an effective marker that can be used to predict and evaluate individuals’ creative performance in the fields of science and art.

## Results

### Behavioral data

Table 1 shows the descriptive statistics of the demographic and psychological characteristics of all participants. No statistically significant differences were found between males and females in terms of the age (mean standard deviation for the males was 20.23 ± 1.34 compared with 19.79 ± 1.26 for the females, *p* = 0.520) or the IQ (CRT score) (mean standard deviation for the males was 65.90 ± 3.68 compared with 66.15 ± 3.28 for the females, *p* = 0.324). The art creativity scores also did not significantly differ between the genders; however, scientific creativity showed a significant gender difference (independent *t*-*test, t*(354) = −2.592, *p* < 0.01), which means the males displaying higher scientific creativity scores than the females.

Moreover, a Pearson correlation coefficient was calculated between the gender, age and IQ. The results indicated that there were no correlations between the IQ and age (*r* = −0.03, *p* = 0.626) and the IQ and gender (*r* = −0.04, *p* = 0.515), but the gender and age have significant correlation (*r* = 0.17, *p* = 0.002), which just suggests that the sample is nonuniform in gender. According to all these results, differences in the distributions of gender, age and IQ did not contribute to the GM analysis findings.

### Neuroimaging data

#### GMV of brain regions significantly correlated with artistic creativity

**Figure 1:**
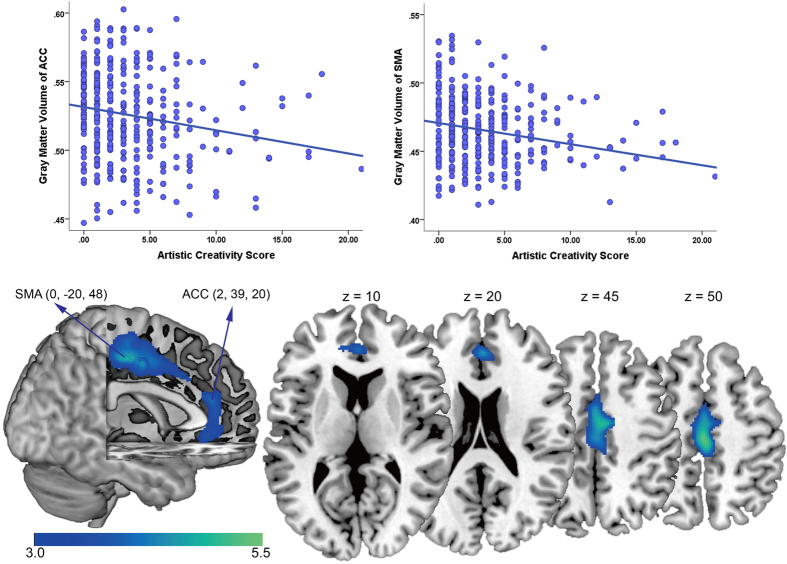
Regions of correlation between GMV and artistic creativity score.

We investigated the association between GMV of brain regions and artistic creativity after controlling age, gender and general intelligence as possible confounds through multiple linear regression analysis. Because correlation analysis suggested that the global GMV was significantly related with scientific creativity (*r* = 0.193, *p* < 0.001), which indicated that the co-variation between whole-brain GMV and creativity could affect the final results, we also take the effect of whole-brain GMV as covariates of no interest. The automated anatomical labeling template^32^ was used to define the brain regions. The results showed that the artistic dimension score of creative achievement was negatively correlated with GMV in the supplementary motor area (BA 6: x, y, z = 0, −20, 48, *t(349)* = −5.59, *p(corr)* < 0.05), ACC (BA 32: x, y, z = 2, 39, 20, *t(349)* = −4.32, *p(corr)* < 0.05). Table 2 and Fig. 1 present the results of the statistical analysis to identify brain areas that are significantly correlated with artistic creativity.

The GMV in two clusters was negatively correlated with the artistic creativity score. Cluster 1 contained the supplementary motor area. Cluster 2 contained the anterior cingulate cortex. The results are shown at *t* > 3 for visualization purposes.

#### GMV of brain regions significantly correlated with scientific creativity

**Figure 2:**
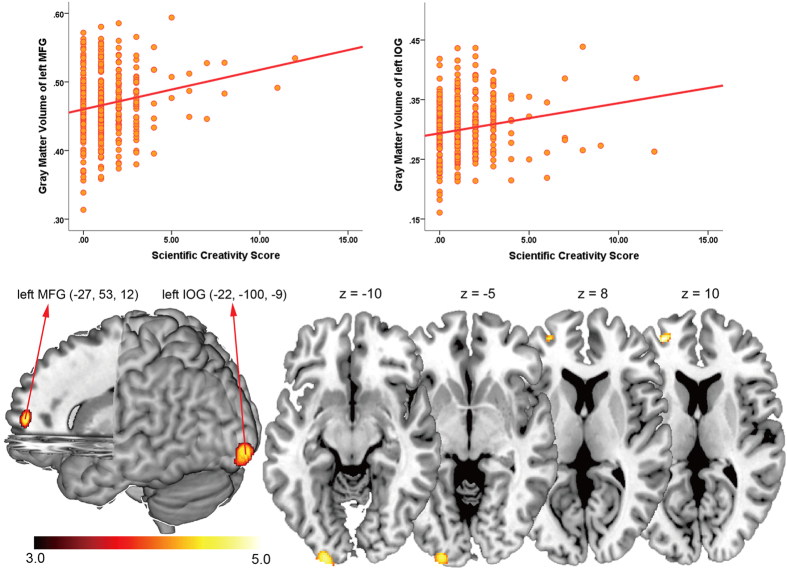
Regions of correlation between GMV and scientific creativity score.

We investigated the association between GMV of brain regions and scientific creativity in the same way just like artistic creativity. The automated anatomical labeling template^32^ was also used to define the brain regions. The results showed that the scientific creative achievement was positively correlated with GMV in left middle frontal gyrus (BA 10: x, y, z = −27, 53, 12, *t(349)* = 4.73, *p(corr)* < 0.05), and left inferior occipital gyrus (BA 18: x, y, z = −22, −100, −9, *t(349)* = 4.52, *p(corr)* < 0.05). Table 3 and Fig. 2 present the results of the statistical analysis to identify brain areas that are significantly correlated with scientific creativity.

The GMV in two clusters was positively correlated with the scientific creativity score. Cluster 1 contained the left middle frontal gyrus, while cluster 2 contained the left inferior occipital gyrus. The results are shown at *t* > 3 for visualization purposes.

## Discussion

The current study explored brain regions associated with artistic and scientific creativity using VBM. In particularly, the results showed that artistic creativity was negatively correlated with GMV in the ACC (the core region of SN) and SMA, whereas scientific creativity was positively correlated with GMV in the MFG and IOG. Some of these results were not consistent with our expectations. Thus, we will discuss the relationship between brain structure and creative achievement and the meaning of this relationship in detail.

The results showed that rGMV in the ACC and SMA were negatively correlated with artistic creativity. Sanfey *et al*.^33^ consider that the ACC takes part in monitoring cognitive conflicts, and its activity may reflect the conflict between cognition and emotion in task processing. Compared to scientists, artists attach greater importance to creating new beauty and to expressing inner desires and emotions^34^, and their negative emotions result in greater artistic creativity^35^. As a consequence, highly artistically creative individuals may experience more emotional conflicts and the ACC can response and solve them. On the other hand, the ACC is a critical node of the SN, and the latter is responsible for evaluating information about the surroundings, for identifying the most relevant reaction, for classifying external stimuli and internal events, and for switching to the relevant processing system. For example, the DMN supports self-related (or internally directed) cognition^36^ while the central executive network supports goal-oriented (or externally directed) cognition^37^. The SN guides appropriate responses to salient stimuli by switching the activation of the DMN and the central executive network^38^. Artistic creativity can be considered a senior cognitive activity because it requires various cognitive abilities, such as sustained attention, the suppression of irrelevant thoughts, working memory and cognitive flexibility, and the SN seems to involve mediating motivated behaviors^39^ and flexibility cognitive control, likely related to individual creative achievement^40^. Hence, we can conclude that the generation of artistic creativity requires the SN. But Bashwiner *et al*.^20^ indicated that musical creativity was implicated in the dorsomedial prefrontal cortex (dMPFC) subsystem of the DMN, which is different with our result about artistic creativity, it is reasonable to assume that this is because musical creativity is only a kind of expression of artistic creativity, which is not equal to artistic creativity itself. However, this different result is worthy to consider in the future study.

Moreover, according to the results of the current study, the temporal lobe is not significantly correlated with artistic creativity. This finding is worthy of discussion because the temporal lobe is correlated with artistic creativity^41^. Previous studies have also indicated that the temporal gyrus is related to divergent thinking^42^, storage and retrieval^43^. The results of this study are thus not consistent with those of previous studies. Importantly, the CAQ measures only existing achievements and not the creative process itself. Differences in the assessment tools employed may be responsible for the differences between the present results and those of previous studies. Future studies need to improve the assessment tools available for artistic creativity and further explore the neural basis of artistic creativity.

The results showed that rGMV in the left MFG and left IOG was positively correlated with scientific creativity. The left MFG may be responsible for integrating loosely or remotely associated semantic concepts into creative ideas^44^. It also plays a critical role in the types of divergent semantic processing that are related to creativity^45^ and is considered to be associated with creative achievement^26^. Overall, the MFG is involved in semantic processing and reasoning. Therefore, highly scientifically creative individuals tend to think and imagine deeply and to be self-critical. Moreover, Andersson *et al*.^46^ suggested that the left middle frontal cortex is part of an executive attention network, maybe we can conclude that scientific creativity involves executive attention network. On the other hand, approximately 80% of sensory information is visual^23^, and the occipital gyrus is a major visual area. Semantics, pronunciation and glyphs are three elements of symbolic language. Semantic information is processed most comprehensively, and semantically related words are closely correlated in visual channels. Therefore, the occipital gyrus is involved in semantic processing^47^. Scientific activities follow more logical rules and involve a lot of abstract semantic reasoning, which may partially explain the significant correlation between scientific creativity and the IOG. Overall, scientific creativity was correlated with the MFG (which is part of the executive attention network) and the IOG (which is involved in semantic processing). As suggested in the introduction and discussion above, highly scientifically creative individuals may have stronger semantic understanding and logical reasoning and may pay more attention to details. Of course, other reasons could also account for the association of scientific creativity with the MFG and IOG. The neural mechanisms of scientific creativity should be revealed by more systematic studies.

In this study, we also found that the cingulate and parietal cortex were not significantly correlated with scientific creativity. This finding is also worthy of discussion because studies have indicated that scientific creativity is closely related to the frontal, parietal and cingulate cortices^27^. Scientific activities follow more logical rules and involve a lot of abstract reasoning, and the study of reasoning and decision-making have consistently shown that the cingulate cortex take part in logical reasoning and in the weighing of costs and benefits^48^. In other words, scientific creativity involves rigorous reasoning, and this process activates the cingulate cortex. The cingulate cortex, which is part of the limbic system, and the frontal gyrus reflect the processing needs of scientific creativity. As the three-factor anatomical model^41^ notes, the limbic system (including the cingulate) is responsible for novelty seeking and for providing creative drive. We hypothesize that the cingulate cortex and frontal lobe are both involved in scientific creativity, but the role of the frontal gyrus is more ambiguous, whereas activation of the frontal gyrus is more obvious.

Based on the discussion above, the overlapping neural bases between scientific and artistic creativity can be regarded as reflecting universal creativity, whereas the differences in the neural bases can be considered an outward manifestation of the domain-specificity of creativity. Overall, the neural bases of artistic and scientific creativity differ, consistent with the idea that creativity can be divided into two types at the physiological level. Understanding the differences between artistic and scientific creativity is important for improving the creativity of individuals in real life.

Through the use of structural MRI, this study revealed the relationship between rGMV and creative achievement. Moreover, this study divided creativity into artistic and scientific creativity and explored the neural basis of creativity from two main perspectives. The majority of previous studies used a single task to measure creativity, such as verbal creativity, visual creativity, and divergent thinking tasks, whereas this study measured creativity using the CAQ, which is comprehensive. Because the CAQ avoids bias, the results using this measure are more persuasive. However, this study had two limitations. First, it explored the relationship between creative achievements and brain regions but did not identify a causal relationship. Subsequent studies should design task-based MRI experiments and systematically explore the relationship between artistic and scientific creativity and specific brain regions. Second, because the CAQ is a self-reported questionnaire, the participants may have exaggerated or minimized their creative achievements during the evaluation. Hence, this measure of creative achievement does not fully reflect objective creativity. Future studies should choose representative scientists and artists as subjects or improve the assessment tools for different creativity.

## Methods

The current study was performed in accordance with the relevant guidelines and regulations in the Declaration of Helsinki. All procedures were approved by the Southwest University Brain Imaging Center (SWUBIC) Institutional Review Board. All subjects provided written informed consent before completing the series of psychological test and the MRI scans and were paid for their participation.

### Subjects

A total of 365 healthy volunteers participated in the study. Since the segmentation and normalization procedure were processed through automation scripts and not manually, the outlier detection was necessary to ensure that the segmentation quality is acceptable for every subject. A Pearson correlation by comparing degree to which participants were correlated to the averaged smoothed GM was used to perform subject outlier detection. We defined outliers as subjects deviated more than 3% from the mean GM volumes and 9 subjects were removed from the total subjects. Thus the valid sample was comprised of 356 subjects (females = 199). All participants were right-handed and displayed normal psychological, mental and physical characteristics. This study was part of an ongoing project to investigate the relationship between brain imaging, creativity, and mental health^29^,^40^.

### Behavioral Measures

#### Creative Achievement Questionnaire (CAQ)

Carson *et al*.^49^ developed the CAQ to measure individuals’ creative achievements through self-reporting. The CAQ consists of 10 specific domains including painting/sculpture, music, dance, invention and scientific discovery etc. Each domain includes 8 items and participants scored from 0 to 7 for every item. 0 represents the “no achievement”, 1 represents the “training” item, and the other six items ascend in turn. Carson *et al*.^49^ wanted to see if the CAQ would be divided into art and science, so they forced a two-factor solution. The result indicated that factor 1 was regarded as “Arts” including drama, humor, music, painting/sculpture, and creative writing; Factor 2 was regarded as “Science” included invention, scientific discovery, and the culinary arts. In this study, we chose the two-factor solution and divided creativity into artistic creativity and scientific creativity.

#### Assessment of general intelligence

Intelligence affects brain structures^30^. In this study, we using the Combined Raven’s Test-Rural in China (CRT-RC3), which was revised by the Psychology Department of East China Normal University in 1994, in order to controlled this effect. Due to its reliability and validity, the CRT is widely used to test intelligence^50^. The CRT is composed of 72 nonverbal items, and the total score is calculated as the sum of correct items. Participants should be able to complete the CRT in 40 min.

#### Data acquisition

Imaging was performed on a Siemens 3T Trios canner (Siemens Medical Systems, Erlangen, Germany) with 8-channel radio frequency coil in the SWUBIC. High-resolution 3D T1-weighted anatomical images were obtained using a Magnetization Prepared Rapid Acquisition Gradient echo (MPRAGE) sequence with the following parameters: TR/TE = 1900 ms/2.52 ms; inversion time (TI) = 900 ms; flip angle = 9°; FOV = 256 × 256 mm^2^; slices = 176; thickness = 1.0 mm; and voxel size = 1 × 1 × 1 mm^3^.

#### Data processing

The preprocessing was executed with the VBM8 toolbox (http://dbm.neuro.uni-jena.de/vbm) based on SPM8 software (Statistical Parametric Mapping, http://www.fil.ion.ucl/spm/software/spm8). Firstly, MR images from each participant were manually reoriented to the anterior commissure for better registration. Then, the images were segmented into gray matter (GM), white matter (WM), and cerebrospinal fluid (CSF) using the new segmentation tool. The preprocessing in this study includes bias correction for image intensity non-uniformity due to the MRI process. After segmentation, we performed image registration, normalization, and modulation using Diffeomorphic Anatomical Registration through Exponentiated Lie (DARTEL) in SPM8^51^. To ensure that regional differences in the absolute amount of gray matter were conserved, each voxel was modulated by Jacobian determinants derived from spatial normalization^52^.Subsequently, the registered images were transformed to Montreal Neurological Institute (MNI) space. Finally, the gray and white matter map of each subject were warped using their corresponding smooth, reversible deformation parameters to the custom template space and then to the MNI standard space. As for GMV and WMV, the warped images of gray and white matter were modulated by calculating the Jacobian determinants derived from the special normalization step and by multiplying each voxel by the relative change in volume^52^. The modulation step was carried out to correct any volume changes during nonlinear normalization. The warped modulated images of gray and white matter were smoothened through the convolution of a 10-mm full-width at half-maximum isotropic Gaussian kernel.

GMV data was analyzed in SPM8. We identify the GMV of brain regions referred to artistic creativity and scientific creativity by performing multiple linear regressions. Age, gender, general intelligence and whole-brain GMV were controlled as possible confounds. An explicit masking generated by the masking toolbox was used to avoid edge effects around the borders between gray and white matter. This approach reduced the risk of false negatives caused by overly restrictive masking, as potentially interesting voxels may be excluded from the statistical analysis^53^.For all analyses, statistical significance was set at a significant level of *p* < 0.05 and corrected at the non-stationary cluster correction^54^ with an underlying voxel level of *p* < 0.001.

## Additional Information

**How to cite this article**: Shi, B. *et al*. Different brain structures associated with artistic and scientific creativity: a voxel-based morphometry study. *Sci. Rep.* **7**, 42911; doi: 10.1038/srep42911 (2017).

**Publisher's note:** Springer Nature remains neutral with regard to jurisdictional claims in published maps and institutional affiliations.

## Figures and Tables

**Table 1:** Descriptive statistics of participants’ demographics and behavioral measures (*N* = 356; males = 157, females = 199).

Measure	Mean	SD	Range
Age	19.98	1.31	17–27
CRT	66.03	3.46	49–72
Artistic creativity	3.72	4.61	0–48
Scientific creativity	1.45	2.07	0–25

*Note*: CRT, Combined Raven’s Test.

**Table 2:** Brain regions whose gray matter volume is significantly correlated with artistic creativity.

Region	*BA*	*MNI coordinates*			*Cluster size*	
		*X*	*Y*	*Z*	*k(voxels)*	*t-score*
Positive correlation	no					
Negative correlation						
Supplementary motor area	6	*0*	*−20*	*48*	3132	−5.59
Anterior cingulate cortex	32	*2*	*39*	*20*	1580	−4.32

*Note: BA* = *Brodmann area. MNI* = Montreal Neurological Institute, voxel size = 1 mm × 1 mm × 1 mm, k = a minimum cluster size; All T-scores reflect a VBM threshold of p < 0.05 (FDR-corrected) and k > 20.

**Table 3:** Brain regions whose gray matter volume is significantly correlated with scientific creativity.

Region	*BA*	*MNI coordinates*			*Cluster size*	*t-score*
		*X*	*Y*	*Z*	*k(voxels)*	
Positive correlation						
Left middle frontal gyrus	10	*−27*	*53*	*12*	132	4.73
Left inferior occipital gyrus	18	*−22*	*−100*	*−9*	148	4.52
Negative correlation	no					

*Note: BA* = *Brodmann area. MNI* = Montreal Neurological Institute, voxel size = 1 mm × 1 mm × 1 mm, k = a minimum cluster size; All T-scores reflect a VBM threshold of p < 0.05 (FDR-corrected) and k > 20.
